# Psoriasis treatment and biologic switching: The association with clinical characteristics and laboratory biomarkers over a 13‐year retrospective study

**DOI:** 10.1111/1346-8138.17465

**Published:** 2024-09-13

**Authors:** Asumi Koyama, Lixin Li, Toyoki Yamamoto, Haruka Taira, Eiki Sugimoto, Yukiko Ito, Yuka Mizuno, Kentaro Awaji, Shoko Tateishi, Hiroko Kanda, Shinichi Sato, Sayaka Shibata

**Affiliations:** ^1^ Department of Dermatology, Graduate School of Medicine The University of Tokyo Tokyo Japan; ^2^ Department of Allergy and Rheumatology, Graduate School of Medicine The University of Tokyo Tokyo Japan; ^3^ Division for Health Service Promotion University of Tokyo Tokyo Japan; ^4^ Immune‐Mediated Diseases Therapy Center, Graduate School of Medicine University of Tokyo Tokyo Japan

**Keywords:** biologics therapy, drug survival, treatment patterns, treatment switching

## Abstract

The advent of biologics has greatly improved patient outcomes, yet some patients are compelled to switch therapies. Predicting these therapeutic failures is important; however, the factors associated with switching biologics have not been fully explored. This study examined patterns and determinants of biologics switching in psoriasis treatment retrospectively over 13 years. We focused on the association between clinical characteristics, basal laboratory data, and frequency of biologics switching. The findings revealed that elevated Psoriasis Area Severity Index scores and the presence of arthritis were observed in patients who experienced two or more treatment switches compared with those without treatment switches. Moreover, neutrophil to lymphocyte ratio was associated with higher biologics switching rates, indicating that systemic inflammation significantly impacts treatment adherence. A treatment approach, taking into account both the clinical presentation and inflammatory biomarkers, may be important for optimizing patient management in psoriasis.

## 1 INTRODUCTION

Psoriasis is a chronic inflammatory skin disease that profoundly impacts patients' quality of life.^1^, ^2^ Symptoms such as visible skin appearance changes, itching, and joint pain can affect both mental and social aspects.^2^, ^3^ In recent years, the advent of biologics has enabled many patients to achieve remission or maintain low disease activity.^4^ Nonetheless, a subset of patients may need to change their biologic therapy because of primary or secondary ineffectiveness or adverse events. Predicting these therapeutic failures is crucial; however, the risk factors associated with switching biologics have not yet been fully explored.

Patients frequently have comorbidities, including arthritis and metabolic syndrome, which are known to be associated with refractory psoriasis.^5^, ^6^, ^7^ These patients are often associated with systemic inflammation, and biomarkers such as C‐reactive protein (CRP) and erythrocyte sedimentation rate (ESR) are used to assess systemic inflammation.^8^, ^9^ These biomarkers, along with hematological ratios such as the neutrophil to lymphocyte ratio (NLR), monocyte to lymphocyte ratio (MLR), and platelet to lymphocyte ratio (PLR) have been identified as practical markers of predicting disease activity in various conditions, including psoriasis, cancers, infectious diseases, and collagen diseases.^10^, ^11^, ^12^, ^13^, ^14^, ^15^, ^16^

Therefore, the purpose of this study is to elucidate the association between clinical characteristics and laboratory data with the necessity of switching biologics in psoriasis treatment. The study also involves a comprehensive analysis of the usage patterns of biologics, including the frequency and trends of switching, in a single‐center setting.

## 2 METHOD

### 2.1 Patients, clinical assessments, and data collection

The present study retrospectively reviewed patients diagnosed with psoriasis vulgaris (PsV), psoriatic arthritis (PsA), or generalized pustular psoriasis (GPP) and treated with biologics at the University of Tokyo Hospital in Tokyo, Japan. The review period spanned from January 2010 to October 2023. This study included patients who initiated their first‐line biologic therapy between January 2010 and November 2021, and those who were observed for more than 24 weeks were enrolled.

Patients were categorized based on the total number of bio‐switches: none, one, or two or more. Baseline information included age, sex, body mass index (BMI), and disease duration before the initiation of biologics. Laboratory data CRP, ESR, NLR, MLR, and PLR were collected within the 3 months preceding the assessment date. The presence of clinical comorbidities, specifically arthritis, diabetes, hypertension, and dyslipidemia was also investigated. Disease severity was assessed by a dermatologist using Psoriasis Area Severity Index (PASI) scores, and arthritis was diagnosed by rheumatologists based on the Classification Criteria for Psoriatic Arthritis (CASPAR) criteria.

The medical ethics committee of the University of Tokyo approved all described studies (No. 3360), and the study was conducted according to the principles of the Declaration of Helsinki.

### 2.2 Statistical analysis

Descriptive statistics are summarized using median and interquartile range (IQR) for continuous variables and absolute numbers with percentage for categorical variables. The Kruskal‐Wallis test with Dunn–Bonferroni post hoc test was conducted for analyzing continuous variables; Fisher exact test with Bonferroni adjustment was used for the assessment of categorical variables. The drug‐specific survival rates were estimated by the Kaplan–Meier method, and the differences between each group were assessed by the log‐rank test. *p* < 0.05 was considered statistically significant throughout all of the analyses. Statistical analyses were conducted using Prism 9 (Graph Pad Software) and EZR software program 7.^17^

## 3 RESULTS

### 3.1 Frequency of biologics switching and switching patterns

In the present study, a total of 285 patients undergoing biologics treatment were included in the analysis. Baseline characteristics of all patients are presented in Table 1. The numbers of patients with PsV, PsA, and GPP were 135 (47.4%), 137 (48.1%), and 13 (4.6%), respectively. These included patients who maintained their initial biologic therapy (*n* = 125; 43.9%), those who underwent a single switch (*n* = 82; 28.8%), those who switched biologics twice (*n* = 42; 14.7%), and those who switched three or more times (*n* = 36; 12.6%). We then focused on 160 patients who had experienced at least one switch between biologics. These agents were categorized into three groups based on their target factors: tumor necrosis factor inhibitor (TNFi), interleukin 17 inhibitor (IL‐17i), and IL‐23 inhibitor (IL‐23i). The distribution of the switching patterns among these biologics is listed in Table 2. All multiple switches per patient were counted for patients who switched biologics twice or more. Switches to Janus kinase (Jak) inhibitors or a tyrosine kinase 2 (Tyk2) inhibitor were excluded. A total of 125 patients maintained their initial biologic therapy and never switched. The number of patients who switched once was 82. The number of patients who switched two or more times was 78, with the breakdown as follows: two switches (42 patients), three switches (19 patients), four switches (11 patients), five switches (five patients), and eight switches (one patient). There were six switches to Jak or Tyk2 inhibitors. Therefore, the total number of switches for patients who changed biologics two or more times was calculated as 2 × 42 + 3 × 19 + 4 × 11 + 5 × 5 + 8 × 1–6 = 212. Adding the 82 single switches, the total number of biologic switches was 294 (82 + 212).

**TABLE 1:** Characteristics of the 285 patients at the initiation of the biologic.

		Value	Range
Total number of patients		285	NA
Initiation age of biologics, median (IQR), years		53 (44–65)	17–84
Men/women, No.		197/88	NA
Subtype of psoriasis, PsV/PsA/GPP		135/137/13	NA
Disease duration, median (IQR), years		14 (6–24)	0–61
PASI, median (IQR)		7.7 (4.3–16.3)	0–48.6
BMI, median (IQR)		24.4 (22.3–28.1)	13.9–65.2
Diabetes (%)		13.3	NA
Hypertension (%)		41.4	NA
Dyslipidemia (%)		22.8	NA

Abbreviations: BMI, body mass index; GPP, generalized pustular psoriasis; IQR, interquartile range; NA, not applicable; PASI, Psoriasis Area and Severity Index; PsA, psoriatic arthritis; PsV, psoriasis vulgaris.

**TABLE 2:** Frequency of biologics switching and switching patterns.

Before switch	After switch	None	Once	Twice	Three times or more	Total
TNFi	TNFi	59	10	3	15	28 (9.5%)
	IL‐17i	NA	25	13	19	57 (19.4%)
	IL‐23i	NA	25	19	18	62 (21.1%)
IL‐17i	TNFi	NA	1	3	10	14 (4.8%)
	IL‐17i	36	3	7	18	28 (9.5%)
	IL‐23i	NA	6	8	15	29 (9.9%)
IL‐23i	TNFi	NA	1	4	9	14 (4.8%)
	IL‐17i	NA	3	11	22	36 (12.2%)
	IL‐23i	30	8	14	4	26 (8.8%)
Total		(125)	82	82	130	294

Abbreviations: IL‐17i, interleukin 17 inhibitor; IL‐23i, interleukin 23 inhibitor; NA, not applicable; TNFi, tumor necrosis factor inhibitor.

The most frequent switching pattern observed was from TNFi to other types, accounting for approximately 50% of the total switches. Switches from IL‐17i and IL‐23i to other drug types were about 25% of the total in both cases. Furthermore, a detailed breakdown of the switching patterns revealed that switching from TNFi to either IL‐17i or IL‐23i was the most common, each accounting for approximately 20% of the total switches. In addition, switching from IL‐23i to IL‐17i constituted approximately 12% of the total, and, vice versa, switching from IL‐17i to IL‐23i accounted for about 10% of the total. Switching between biologics targeting the same factors was less common, with 9.5% for TNFi and IL‐17i and 8.8% for IL‐23i.

### 3.2 Characteristics associated with frequencies of biologics switching

Next, we investigated the association between the number of biologics switches and the baseline patient characteristics before treatment initiation. Patients were categorized into three groups according to the number of biologics switches: none (*n* = 125; 43.9%), one (*n* = 82; 28.8%), and two or more (*n* = 78; 27.4%). The baseline characteristics of these groups are shown in Table 3. Notably, more frequent biologics switches were significantly associated with higher PASI scores at the initiation of biologic therapy, with scores averaging 12.3 (IQR: 4.8–21.9) for patients with two or more switches compared with 6.6 (IQR: 4.15–13.2) for those with no switches (*p* = 0.016, Fisher exact test with Bonferroni adjustment). Furthermore, a higher prevalence of concurrent arthritis at enrollment was observed in patients with two or more switches (64.1%) compared with those with no switches (43.2%; *p* = 0.012, Fisher exact test with Bonferroni adjustment). No significant differences were noted in terms of sex, age at initiation of biologics, disease duration, or BMI among the different groups.

**TABLE 3:** Clinical characteristics of patients categorized by the number of switches.

No. of switches		None	Once	Twice or more	*p* value
Patients, No.		125	82	78	
Sex, men/women, No.		86/39	57/25	54/24	1
Initiation age of biologics, median (IQR), years		54	55	50	0.105
		(45–66)	(45–67)	(42–59)	
Concurrent arthritis at the initiation of biologics, No. (%)		54 (43.2%)	39 (47.6%)	50 (64.1%)	0.012
PASI score, median (IQR)		6.6	9.1	12.3	0.016
		(4.15–13.2)	(4.45–17.85)	(4.8–21.9)	
Disease duration prior to the initiation of biologics, median (IQR), years		14	15	13	0.689
		(7.0–24.0)	(6.25–24.75)	(5.25–24)	
BMI, median (IQR)		24.05	24.57	25.09	0.442
		(22.32–27.04)	(21.61–28.14)	(22.64–28.80)	
Comorbidities, No. (%)	Diabetes	19 (15.2%)	9 (11.0%)	10 (12.8%)	0.681
	Hypertension	56 (44.8%)	37 (45.1%)	25 (32.1%)	0.146
	Dyslipidemia	32 (25.6%)	23 (28.0%)	10 (12.8%)	0.036

Abbreviations: BMI, body mass index; IQR, interquartile range; PASI, psoriasis area and severity index.

Regarding comorbidities, a significant variation was observed in the frequency of dyslipidemia complications among the groups. Patients with two or more switches showed the lowest prevalence of dyslipidemia (12.8%), compared with 25.6% in patients with no switches and 28.0% in those with one switch (*p* = 0.036, Fisher exact test with Bonferroni adjustment). There were no significant differences in the prevalence of diabetes and hypertension between the groups.

### 3.3 Comparisons of laboratory biomarkers between frequencies of biologics switching

We subsequently examined the association between circulating inflammatory parameters and the frequency of biologics switching. Baseline laboratory data for each group are summarized in Table 4. The NLR was 2.65 (IQR: 2.03–3.97) for patients with two or more switches, and 2.55 (IQR: 1.91–3.45) for patients with one switch, which was higher compared with 2.3 (IQR: 1.60–3.27) for those with no switches (*p* = 0.011, Kruskal‐Wallis test with Dunn–Bonferroni post hoc test). Although CRP and ESR levels tended to be higher in patients with two or more switches, these differences did not reach statistical significance. In addition, a difference analysis was also performed for MLR and PLR, but there were no significant differences among the groups. We also investigated the association between higher PASI scores, NLR, and the frequency of biologic switching across different type of psoriasis. The small number of patients with GPP in our study limited our ability for further analysis. Regarding PsA and PsV, PASI scores did not significantly correlate with biologic switching for individual psoriasis types, and a significant association was observed when analyzed as a whole (Table S1). Higher NLR was significantly associated with increased biologic switching in patients with PsA but not in patients with PsV (Table S1).

**TABLE 4:** Baseline laboratory biomarkers categorized by the number of switches.

Number of switches	None	Once	Twice or more	*p* value
Patients, No.	125	82	78	
CRP, median (IQR)	0.12	0.09	0.18	0.517
	(0.05–0.42)	(0.05–0.31)	(0.05–0.57)	
ESR, median (IQR)	13.5	16	24	0.169
	(7–25)	(7–29)	(8.25–37)	
NLR, median (IQR)	2.3	2.55	2.65	0.011
	(1.60–3.27)	(1.91–3.45)	(2.03–3.97)	
MLR, median (IQR)	0.23	0.25	0.25	0.109
	(0.18–0.30)	(0.19–0.36)	(0.09–0.31)	
PLR, median (IQR)	14.18	16.1	15.77	0.137
	(10.98–19)	(12.14–20.94)	(12.42–21.79)	

Abbreviations: CRP, C‐reactive protein; ESR, erythrocyte sedimentation rate; IQR, interquartile range; MLR, monocyte to lymphocyte ratio; NLR, neutrophil to lymphocyte ratio; PLR, platelet to lymphocyte ratio.

### 3.4 Drug survival of biologics stratified by their targets

**FIGURE 1:**
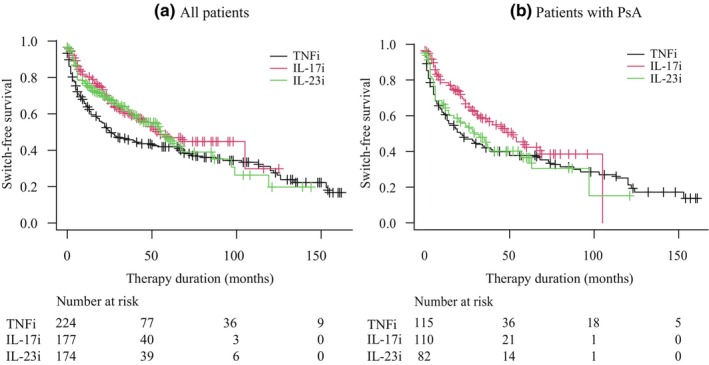
Kaplan–Meier drug survival curves grouped by biologic classes of all patients and patients with psoriatic arthritis (PsA). Interleukin 17 inhibitor (IL‐17i) had a longer drug survival than tumor necrosis factor inhibitor (TNFi) in all patients (*p* = 0.007, a) and in patients with PsA (*p* = 0.033, b). IL‐23i, interleukin 23 inhibitor.

Kaplan–Meier curves were then utilized to analyze the drug survival rates of all patients and those with arthritis (Figure 1). The median overall drug survival of all patients was 25 months for TNFi, 54 months for IL‐17i, and 55 months for IL‐23i (95% confidence interval [CI]: 19–55 for TNFi, 40–NA for IL‐17i, and 41–86 for IL‐23i). We found that IL‐17i and IL‐23i had a longer drug survival than TNFi, with statistically significant differences observed between TNFi and IL‐17i (*p* = 0.007). While the difference between TNFi and IL‐23i did not reach statistical significance, there was a notable trend towards significance (*p* = 0.051).

We also generated a Kaplan–Meier curve for patients with PsA. The median overall drug survival of patients with PsA was 21 months for TNFi, 52 months for IL‐17i, and 29 months for IL‐23i (95% CI: 13–40 for TNFi, 31–NA for IL‐17i, and 14–57 for IL‐23i). A statistically significant difference was found between TNFi and IL‐17i (*p* = 0.033). IL‐23i showed a trend towards shorter drug survival compared with IL‐17i (*p* = 0.088) but was not statistically significant.

## 4 DISCUSSION

In 2017, Honda et al. reported that one‐sixth of patients with psoriasis and arthritis from a cohort of 51 individuals, experienced a switch in biologic therapy.^18^ Since 2015, biologics targeting IL‐17A, IL‐17F, IL‐17RA, and IL‐23p19 have become available in Japan, thus expanding treatment options for patients with psoriasis.^19^ Given this background, this study aimed to investigate long‐term data over 13 years on biologics switching in the treatment of psoriasis and to explore the factors impacting these treatment changes. Despite the broader treatment options, patients with concurrent arthritis exhibited a higher rate of switching biologics in the present study. Furthermore, higher PASI scores were also associated with a higher rate of switches. This observation was supported by the association between an increase in the NLR, a marker of systemic inflammation, and frequencies of changing biologics. Such trends were particularly pronounced in patients who experienced two or more than two switches. These results may indicate a significant impact of systemic inflammation on treatment outcomes and emphasize the necessity for adjustments in therapeutic strategies according to individual patient profiles.

In an evaluation of biologics switching background from the perspective of PsA, it was reported that older age, female sex, arthritis disease status indicator such as Health Assessment Questionnaire and the change in Ankylosing Spondylitis Disease Activity Score, and shorter disease duration were linked to higher rates of switching.^20^, ^21^, ^22^, ^23^, ^24^, ^25^ While association with sex or age was not evident in the present study, the intensity of inflammation is commonly likely to impact the subsequent adherence to treatment.^26^ The coexistence of metabolic syndrome is known to be associated with systemic inflammation; however, it did not directly affect decisions regarding biologic therapy in this study.^27^, ^28^ Instead, the severity of skin and joint symptoms, which are central to the disease's manifestation, may be more influential in determining treatment adherence. Furthermore, NLR was the sole inflammatory marker associated with the frequency of switching, indicating the significance of neutrophil activity in psoriasis pathogenesis and its potential as a critical marker for treatment adherence.^29^, ^30^, ^31^ NLR is recognized as a severity marker in various conditions, including psoriasis. In addition, the formation of neutrophil extracellular traps has been reported to release inflammatory cytokines, indicating that neutrophil activation plays a crucial role in the disease's pathogenesis.^32^, ^33^ Our research aimed to determine a cutoff value for NLR to assess each drug survival in patients with elevated NLR. However, because of the nonnormal distribution of NLR values, establishing an accurate cutoff was not feasible. Further studies with larger sample sizes are necessary to identify which biologics are most effective for patients with a high NLR.

Our drug survival analysis suggested that patients receiving an IL‐17i or IL‐23i were less likely to switch to another biologic than those treated with a TNFi. The lower drug survival rate for TNFi may be affected not only by secondary ineffectiveness and side effects but also by the timing of TNFi approval. A significant number of patients initially treated with a TNFi may have switched to an IL‐17i or IL‐23i after their release because of their high efficacy and safety profiles. In the analysis focusing on PsA, IL‐23i showed a trend towards shorter drug survival compared with IL‐17i, although this difference was not statistically significant. While IL‐23i has established efficacy against peripheral arthritis, the effectiveness against axial arthritis remains unestablished.^34^ Although the present study lacked sufficient information to perform a subanalysis of peripheral and axial arthritis, the findings suggest that in real‐world clinical practice, IL‐17i may be used for longer durations in patients with psoriasis and arthritis.

There are several limitations to the present study. The analysis was performed at a single center. Second, the varied timing of market introduction for drugs used in psoriasis treatment may have influenced a higher switch rate, particularly with TNFi that were available earlier.^19^ Thus, the background of patients initially treated with a TNFi may have influenced the study outcomes. Third, while this study suggests the importance of evaluating several indicators before biologics treatment, it does not recommend the selection of a specific therapeutic target. In future studies, we hope to utilize these biomarkers to establish therapeutic strategies for selecting more appropriate agents in the early stage of treatment.

In summary, the current study demonstrated an association between elevated PASI scores, NLR, and comorbid arthritis and the frequency of biologics switching. A treatment approach, taking into account both the clinical presentation and inflammatory biomarkers, may be important for optimizing patient management in psoriasis.

## CONFLICT OF INTEREST STATEMENT

The authors declare no conflict of interest.

## Supporting information


Table S1.
Table S2.
