# Supplementary material for: Psoriasis treatment and biologic switching: The association with clinical characteristics and laboratory biomarkers over a 13‐year retrospective study
Source: J Dermatol. 2024 Sep 13;51(12):1572–8. doi: 10.1111/1346-8138.17465 (PMC11624150; doi:10.1111/1346-8138.17465)
Supplement: Supplementary file 1 — Table S1. Table S2. [file JDE-51-1572-s001.docx]

**supplementary Table 1. Characteristics of the PsV patients at the initiation of the biologic**

| Number of switches | | None | Once | Twice or more | *P* value |
| --- | --- | --- | --- | --- | --- |
| Patients, No. | | 67 | 41 | 27 |  |
| Sex | M/F, No. | 48/19 | 31/10 | 20/7 | .966 |
| The initiation age of biologics, years (median, IQR) | | 52 | 51 | 45 | .072 |
|  |  | (41.5-63) | (43-62) | (40.5-50.5) |  |
| PASI score (median, IQR) | | 6.6 | 9.7 | 10.65 | .186 |
|  |  | (4.4-13.2) | (5.9-15.0) | (4.9-19.0) |  |
| Disease duration prior to the initiation of biologics, years (median, IQR) | | 11 | 13 | 11 | .976 |
|  |  | (6-24) | (5-24.8) | (6.8-19.5) |  |
| BMI (median, IQR) | | 23.7 | 25.7 | 25.3 | .351 |
|  |  | (21.8-26.0) | (21.7-29.6) | (21.8-28.3) |  |
| Comorbidities, No., (%) | DM | 7 (10.4) | 5 (12.2) | 3 (11.1) | .681 |
|  | HT | 28 (41.8) | 21 (51.2) | 7 (25.9) | .146 |
|  | DL | 17 (25.4) | 14 (34.1) | 3 (11.1) | .036 |
| CRP (median, IQR) | | 0.10 | 0.08 | 0.07 | .763 |
|  |  | (0.04-0.22) | (0.04-0.15) | (0.04-0.19) |  |
| ESR (median, IQR) | | 11 | 13.5 | 21 | .204 |
|  |  | (6-19) | (6-22.8) | (9.3-34.3) |  |
| NLR (median, IQR) | | 2.3 | 2.2 | 2.6 | .639 |
|  |  | (1.6-3.2) | (1.8-3.1) | (1.9-3.2) |  |
| MLR (median, IQR) | | 0.22 | 0.24 | 0.23 | .614 |
|  |  | (0.18-0.29) | (0.18-0.33) | (0.21-0.30) |  |
| PLR (median, IQR) | | 14.1 | 15.0 | 14.8 | .802 |
|  |  | (11-18.6) | (11.7-18.7) | (11.3-18.9) |  |

Abbreviations: M, male; F, female; IQR, interquartile range; PASI, Psoriasis Area and Severity Index; BMI, body mass index; DM, diabetes mellitus; HT, hypertension; DL, dyslipidemia; CRP, C-reactive protein; ESR, erythrocyte sedimentation rate; NLR, neutrophil-to-lymphocyte ratio; MLR, monocyte-to-lymphocyte ratio; PLR, platelet-to-lymphocyte ratio

**supplementary Table 2. Characteristics of the PsA patients at the initiation of the biologic**

| Number of switches | | None | Once | Twice or more | *P* value |
| --- | --- | --- | --- | --- | --- |
| Patients, No. | | 53 | 37 | 47 |  |
| Sex | M/F, No. | 36/17 | 24/13 | .973 | .973 |
| The initiation age of biologics, years (median, IQR) | | 55 | 57 | 53 | .408 |
|  |  | (49-67) | (47-68) | (43.5-65) |  |
| PASI score (median, IQR) | | 7.1 | 7.4 | 13.9 | . 059 |
|  |  | (3.7-14.0) | (3.4-21.2) | (6.3-23.3) |  |
| Disease duration prior to the initiation of biologics, years (median, IQR) | | 15 | 18 | 13 | .578 |
|  |  | (9-22) | (7-23.5) | (4.5-25.5) |  |
| BMI (median, IQR) | | 25.2 | 24.4 | 25.2 | .274 |
|  |  | (22.6-29.3) | (22.6-26.0) | (22.6-29.0) |  |
| Comorbidities, No., (%) | DM | 12 (22.6) | 2 (5.4) | 6 (12.8) | .074 |
|  | HT | 27 (50.9) | 14 (37.8) | 18 (38.3) | .362 |
|  | DL | 14 (26.4) | 8 (21.6) | 7 (14.9) | .378 |
| CRP (median, IQR) | | 0.21 | 0.16 | 0.20 | .761 |
|  |  | (0.08-1.00) | (0.06-0.85) | (0.07-0.58) |  |
| ESR (median, IQR) | | 14 | 21 | 22 | .626 |
|  |  | (10-33) | (12-33.5) | (6-32) |  |
| NLR (median, IQR) | | 2.23 | 2.84 | 2.65 | **.024** |
|  |  | (1.59-3.29) | (2.11-3.42) | (2.13-4.02) |  |
| MLR (median, IQR) | | 0.23 | 0.24 | 0.27 | .212 |
|  |  | (0.17-0.31) | (0.19-0.37) | (0.21-0.33) |  |
| PLR (median, IQR) | | 14.3 | 18.1 | 16.3 | .142 |
|  |  | (10.9-19) | (13-22.6) | (12.5-21.7) |  |

Abbreviations: M, male; F, female; IQR, interquartile range; PASI, Psoriasis Area and Severity Index; BMI, body mass index; DM, diabetes mellitus; HT, hypertension; DL, dyslipidemia; CRP, C-reactive protein; ESR, erythrocyte sedimentation rate; NLR, neutrophil-to-lymphocyte ratio; MLR, monocyte-to-lymphocyte ratio; PLR, platelet-to-lymphocyte ratio
